# Delineating Purinergic Signaling in *Drosophila*

**DOI:** 10.3390/ijms232315196

**Published:** 2022-12-02

**Authors:** Cinzia Volonté, Francesca Alberti, Giuseppe Vitale, Francesco Liguori

**Affiliations:** 1 National Research Council, Institute for Systems Analysis and Computer Science “A. Ruberti”, Via Dei Taurini 19, 00185 Rome, Italy; 2 IRCCS Santa Lucia Foundation, Cellular Neurobiology Unit, Via Del Fosso di Fiorano 65, 00143 Rome, Italy

**Keywords:** adenosine receptor, adenosine deaminase, *Drosophila*, nucleoside transporter, P2X2 receptor

## Abstract

Simplistic models can aid in discovering what is important in the context of normal and pathological behavior. First recognized as a genetic model more than 100 years ago, to date, fruit flies (*Drosophila melanogaster*) still remain an astonishingly good laboratory stand-in for scientists to study development and physiology and to investigate the molecular mechanisms of human diseases. This is because fruit flies indeed represent a simplistic model. Furthermore, about 75% of human disease-related genes have their counterparts in the *Drosophila* genome, added to the fact that fruit flies are inexpensive and extremely easy to maintain, being invertebrates and, moreover, lacking any ethical concern issues. Purinergic signaling is, by definition, mediated by extracellular purinergic ligands, among which ATP represents the prototype molecule. A key feature that has progressively emerged when dissecting the purinergic mechanisms is the multilayer and dynamic nature of the signaling sustained by purinergic ligands. Indeed, these last are sequentially metabolized by several different ectonucleotidases, which generate the ligands that simultaneously activate several different purinergic receptors. Since significant purinergic actions have also been described in *Drosophila*, the aim of the present work is to provide a comprehensive picture of the purinergic events occurring in fruit flies.

## 1. *Drosophila* Made to Model and Understand

When you decide to use *Drosophila melanogaster*, or fruit fly, for understanding and modeling normal and/or disease-related functions and conditions, you might encounter disappointment and skepticism. Without doubt, someone might inquire: what does a fruit fly, a small yet sophisticated organism, have to do with pathophysiology in humans? While justified at first sight, conversely, we trust that using simplistic models can truly aid in discovering what is important in the context of normal and diseased behavior. Indeed, fruit flies are an extremely simple but powerful model for dissecting functions and predicting defects that occur in higher organisms, including human beings [1]. This is because: (a) the entire *Drosophila* genome is sequenced and almost fully annotated [2,3], (b) the heritable mutants of thousands of fruit flies are already characterized, (c) about 75% of human disease-related genes have their counterparts in the fruit fly genome [4], and (d) finally, various gene-manipulating and gene-editing approaches have been developed in *Drosophila* for the functional analysis of human transgenes [5]. In other words, *Drosophila* provides researchers with the most sophisticated but straightforward molecular and genetic tools to determine with no trouble exactly what, how, where, and when things might go right or wrong in the organism. Last, but not least, by representing the state-of-the-art approach for genetic targeting and manipulation of all higher eukaryotes, *Drosophila* allows us to investigate with unprecedented accuracy the complexity of gene networks, the transcriptional roadmap of development, and the molecular mechanisms underlying biological processes up to the high-level intricacy of the nervous system, for instance. Thus, fruit flies are an astonishingly good laboratory stand-in for scientists to study physiology and pathology [6].

A comprehensive description of *Drosophila*’s anatomical, physiological, and behavioral features is out of the scope of the present work. For a brief summary, please refer to Appendix A or to recent publications on the topic by distinguished scientists.

## 2. Benefits, Barriers, and Uncertainties in *Drosophila* Research

**Benefits.** *Drosophila* is an extremely easy to rear animal model. The flies do not require dedicated animal facilities but only marginal logistic and inexpensive maintenance efforts; moreover, they lack ethical concern issues, being invertebrates. Importantly, they are very prolific even in captivity, lying about 100 eggs/day/female, with a quite rapid embryogenesis completed within approximately 24 h after fertilization, and an entire life cycle from embryo, larva (4 days), pupa (4 days), to adult lasting almost 90 days, although variable as a function of the environmental temperature [7]. As additional advantages, there are multiple strategies utilized for gene targeting in *Drosophila*, comprising ethyl methanesulfonate mutagenesis, transposable element insertion, homologous recombination (insertional, replacement), site-specific nuclease systems, such as zinc-finger nuclease or transcription activator-like effector nuclease and, not least, the CRISPR/Cas9 genome editing system [8]. Moreover, multiple methods for drug delivery can be adopted in *Drosophila* trials, such as by feeding, injection, and inhaling, overall, allowing a large repertoire of compounds with different molecular structures and solubility properties to be promptly tested. Furthermore, fruit flies can be securely anesthetized. Finally, a number of diverse morpho-functional parameters can be dissected with no problem in fruit flies while modeling pathophysiological functions. These comprise larval crawling, eclosion rate, adult fly climbing, lifespan, and phenotypical and molecular derangements.

**Barriers.** Some limitations, however, exist concerning full exploitation of *Drosophila* for research purposes. For instance, major organs comprising the brain are quite different in flies and in humans, the adaptive immune system has no counterpart in flies, and only superficial cognitive abilities are manifest in fruit flies. These barriers compromise, at least in part, the indiscriminate use of these nevertheless versatile organisms. 

**Uncertainties.** Despite the numerous advantages and few impediments reported above, the effects of some drugs are also described to be divergent between flies and humans. This notion generates some reservations in the use of *Drosophila* from time to time, thus always requiring comparative screening in more evolved animal models, for instance, and particularly, when assessing preclinical drug development. 

## 3. An Ensemble Approach to Purinergic Signaling 

Purinergic signaling is the one mediated by extracellular purinergic ligands, among which ATP is considered the prototype molecule. Of note, the field has posed its milestone on a seminal discovery made about 50 years ago by *G. Burnstock*, establishing that “exactly ATP was the transmitter extracellularly released by non-adrenergic inhibitory nerves in the gut” [9]. Since then, a huge amount of information has flooded the field and improved our purinergic knowledge [10]. Of particular importance, medicinal chemistry has generated a great number of purinergic agonists/antagonists with high affinity and selectivity for the different variants of the existing purinergic receptors. These have much aided in the understanding of purinergic signaling and mechanisms. As a consequence, numerous purinergic drugs are nowadays under investigational trials, or approved, for several clinical indications [11,12,13].

A key feature that has progressively emerged when dissecting and interpreting purinergic mechanisms is the multilayer and dynamic nature of the signaling sustained by purinergic ligands [14]. To better clarify this concept, we should for instance keep in mind that “dynamic cooperative signaling” is necessarily originated when ectonucleotidases (the ectoenzymes hydrolyzing nucleoside 5′-tri-, 5′-di-, and 5′-mono-phosphates) catabolize the agonists for P2 receptors while, at the same time and within the same chemical reaction, they synthetize the agonists for P1 receptors. Dynamic cooperative signaling further occurs when multiple receptor subtypes are expressed on a given cell phenotype and when the same purinergic agonist or antagonist binds to more than a single receptor subtype, although within different affinity binding properties. As a consequence, multiple receptors are actively engaged by purinergic ligands at the same time on the same cell, and the overall response is precisely the summation of these different molecular interactions [15]. In other words, union makes strength. However, the need of reinforcing any key biological response by the redundancy of receptors or ligands is surely not the only explanation for clarifying cooperative purinergic signaling. Indeed, the one-to-one purinergic receptor–function correlation is nowadays overcome by the newly “purinergic network” concept, according to which the entire pattern of purinergic ectonucleotidases, receptors, transporters, and ligands that work in synergy should be taken into account when trying to decode a more comprehensive purinergic signaling [16]. 

A short description of the existing different classes of purinergic players is provided in Appendix B. 

## 4. Underpinning Purinergic Signaling in *Drosophila*

Within this new inclusive view of the purinergic network signaling, we can easily recognize that various purinergic actions have also already been described in *Drosophila*. Large evidence indeed supports the existence of purinergic transmission in fruit flies, and particularly the one mediated by adenosine, a widespread metabolite acting as a paracrine homeostatic signal of metabolic and additional stress stimuli within tissues, while also regulating energy metabolism and controlling cell growth and survival. 

**Figure 1:**
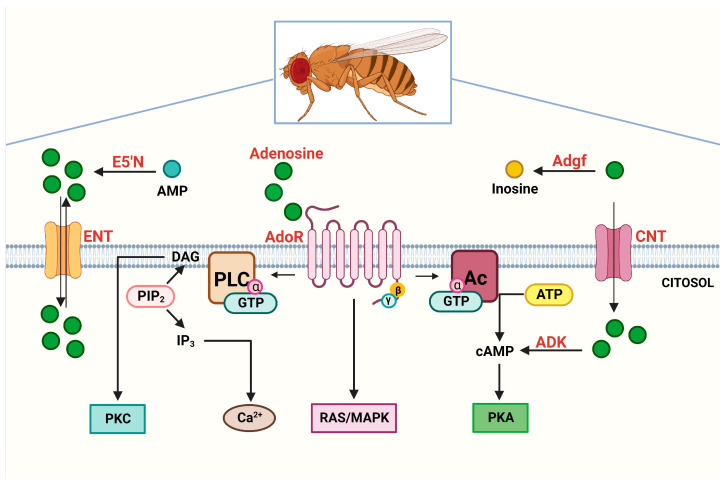
Adenosine signal transduction in *Drosophila*. AdoR: Adenosine Receptor; ENT: Equilibrative Nucleoside Transporter; CNT: Concentrative Nucleoside Transporter; e5′N: ecto-5′-nucleotidase; Adgf: Adenosine Deaminase; ADK: Adenosine Kinase. Figure 1 was created with BioRender.com.

The integrative picture of the extracellular roles of adenosine in *Drosophila* comprises the synergistic action of a single Adenosine Receptor (AdoR), of Equilibrative Nucleoside Transporters (ENT1-3, bidirectional carriers embedded in the biological membrane), of Concentrative Transporters (CNT1-2, unidirectional ATP-consuming carriers acting against the gradient of adenosine concentration), of ecto-5′-nucleotidases (ectoenzymes hydrolyzing nucleoside 5′-mono-phosphates), and of Adenosine Deaminases (Adgf A-E, ectoenzymes interacting with cell surface receptors and acting as co-stimulatory, regulatory proteins to facilitate adenosine signaling) (Figure 1).

### 4.1. Evidence about Adenosine Receptors

The first work establishing a certain involvement of adenosine in *Drosophila* goes back to 1998, when Riegel and coauthors established that external application of adenosine (neither AMP, nor ATP) significantly stimulates the fluid secretion rate from Malpighian tubules (the main osmoregulatory and excretory organs of insects) isolated from *Drosophila*, although to a lesser extent than 3′,5′-cyclic monophosphates of inosine, cytidine, uridine, and thymidine [17]. However, the effect is slow to develop, small in magnitude, selective for some tubules, and, apparently, not mediated by receptors but by a carrier-based transport mechanism. The authors did not exclude the possibility that Malpighian tubes express a membrane receptor responsible for fluid secretion that was not yet identified at that time [17]. 

As a matter of fact, the *CG9753* gene of the fruit fly was only later reported as the first invertebrate AdoR to be decoded [18], sharing homology with vertebrate AdoR and approximately 38% identity with 350 amino acids in the N-terminal region of the *ADORA2A* human receptor [19]. However, differently from mammals, *Drosophila* possesses a single AdoR involved in neuromodulation and responses to stress, positively coupled to adenylate cyclase and resembling an A2A receptor [20]. Transcriptional activation of *CG9753* was found in various *Drosophila*s’ developmental stages and tissues, for instance in brain, imaginal discs, ring and salivary glands, in both larval and adult organisms. Moreover, overexpression of *CG9753* in vivo in *Drosophila* causes developmental abnormalities and lethality, with an increase in intracellular cAMP and calcium levels. On the other hand, *Drosophila* AdoR mutants are viable and show no overt phenotype [19,21]. Overall, these results demonstrate not only that the *CG9753* gene encodes a functional AdoR, but also that AdoR constitutes an essential part of adenosine signaling in fruit flies. In particular, stress, immune, and sleep-waking responses are described among the functions sustained by extracellular adenosine and likely connected to the endogenous expression of AdoR in *Drosophila*’s brain [22,23]. However, when chronically provided to the fruit flies, the adenosine antagonist and psychoactive substance caffeine lengthens the circadian period and induces the wake-promoting effect of reduced and fragmented sleep. This occurs not by antagonism at AdoR (similar effects are obtained in both control and AdoR null flies), but rather by elevation of cAMP levels and activation of protein kinase A [21]. Indeed, the pan-neuronal inhibition of protein kinase A in wild-type flies suppresses the effects of chronic caffeine administration in *Drosophila* [21].

Multipotent intestinal stem cells (ISCs) populate the *Drosophila* midgut epithelium and are strictly dependent on their microenvironment for proliferation or differentiation into enteroblasts (EBs), i.e., progenitor cells in turn primed for differentiation into enterocytes (ECs, the major cell type in number) or enteroendocrine cells (EEs). Precise control of ISCs’ activity is crucial for instance for cellular homeostasis and tumor prevention. A recent RNA interference study has identified 350 *Drosophila* genes which are orthologous to human genes and encode transmembrane and nuclear receptors implicated in ISCs regulation [24]. AdoR was identified as a top candidate receptor required to maintain the ISCs/EB cellular pool, because *AdoR* RNAi as well as sgRNA-directed *AdoR* knockout in ISCs/EB (not in ECs or EEs) have underlined a proliferation defect exclusively emerging under tissue-damaging conditions. Conversely, AdoR overexpression in ISCs/EBs cells induces ISC proliferation. As AdoR downstream mechanisms, protein kinase A and Ca2+/Ras/MAPK were identified to regulate ISC proliferation activity [24]. 

Disturbance of epithelial integrity and apico-basal polarity participates in chronic inflammatory conditions by activating danger signals, among which is the release of adenosine into the extracellular space through equilibrative channels. This can also occur under low-level homeostasis imbalance. During sub-apoptotic chronic perturbation of polarity in epithelial cells, the release of adenosine turns into a warning signal to activate AdoR, in turn behaving as an upstream transcriptional activator of tumor necrosis factor to boost the Jun N-terminal kinase signaling [25]. However, extracellular adenosine is unlikely to act as a wide range warning signal in epithelial cells, because it is short-lived. For this reason, it was suggested that a large number of contiguous epithelial cells have to be engaged to collectively release enough adenosine to ensure a stress response. This model of a “private inflammatory-like response without the involvement of immune cells” is in contrast with the common view that activated monocytes and macrophages within the epithelial damaged tissue produce tumor necrosis factor that is generally inhibited by adenosine [25].

### 4.2. Evidence about Adenosine Transporters and Metabolic Enzymes

As anticipated above, nucleoside transporters [26,27] and adenosine deaminases [28,29] modulate the concentration of adenosine as a signaling molecule and, in addition to AdoR, show substantial conservation (24–33% sequence homology) and topology similarity in *Drosophila* and humans, despite limited orthologue protein sequence identity [30]. 

In particular, *Drosophila* presents five genes with eight alternative transcripts encoding proteins with sequence homology to mammalian ecto-5′-nucleotidases and converting extracellular AMP to adenosine, two of which are GPI-linked proteins with extracellular ecto-5′-nucleotidase activity, although functioning also as soluble released proteins. Interestingly, these two proteins (NT5E-1, NT5E-2) show the highest amino acid sequence similarity to the human ec-to-5′-nucleotidase CD73 [31].

Despite the recognized expression of ecto-5′-nucleotidases in flies, the presence or presumed mechanism of transport and accumulation of extracellular AMP have never been investigated in tissues or in hemolymph from *Drosophila* so far, unlike what has been reported, for instance, from mussels [32].

Concentrative transporters possess restricted and specialized tissue distribution within organisms. In flies, CNT1 and CNT2 activities are generally associated to the toxic effects exerted by high concentrations of extracellular adenosine. Ablation of these proteins accordingly rescues cell death, morphological changes, and mitochondrial polarity, for instance, in cells of *Drosophila* imaginal discs (larval epithelial structures originating during embryonic development from ectodermal cells to differentiate into legs, wings, and head in the adult insect) [33]. The expression of the *Cnt1* gene is restricted to testis in the adult fly, and a very recent work has established that its mutation causes defective mating behavior, abnormal spermatid tail, low sperm count, and male infertility [34].

Equilibrative *Ent2* null mutants are fatal during larval and early pupal stages, suggesting that ENT2 transporters are required for development, while hypomorphic mutant alleles are viable, although presenting reduced associative learning. Excitatory junction potentials and stimulus-dependent calcium influx in presynaptic terminals are potentiated in *Ent2* mutants, while paired-pulse plasticity is reduced [27]. Interestingly, compensatory changes exist in *Drosophila* in the expression of the ENT2 transporter in AdoR mutants, as well as in the expression of adenosine deaminase-related growth factor-A protein (ADGF-A) in *Ent2* mutants [27]. Intriguingly, recent evidence shows that reduced adenosine signaling is protective in a fly model of Huntington’s Disease. In particular, *AdoR* and *Ent2* functional inactivation ameliorates oxidative and heat-shock stress tolerance in mutant Huntingtin-expressing flies [35]. This offers important insights about adenosine-mediated stress response in *Drosophila*.

*Adgf-A* is a gene homologue to human *Cecr1* belonging to the family of adenosine deaminase 2-*Ada2* genes, strongly expressed in the gut and lymph glands, causing polarization and serum-independent proliferation of imaginal disks and embryonic cells in vitro. Similarly to the null *Ent2* mutant, the mutant *Adgf-A*, causing elevated amounts of extracellular adenosine and inhibiting proliferation, is lethal in homozygosis [23,36]. Genetic manipulations of *AdoR*, *Ent2*, and *Adgf-A* altogether influence the signaling of adenosine and cause remarkable changes in the frequency of hyperplastic outgrowth by the loss of heterozygosity of the tumor suppressor gene *warts* in the epithelium of imaginal discs, neural epithelium, and histoblasts nests [37], thus implying a synergistic action directly and/or indirectly exerted by *AdoR*, *Ent2*, and *Adgf-A* at multiple levels. 

Overall, these results demonstrate the broad range of cytotoxic/growth promoting or immunosuppressive actions induced by the modulation of adenosine signaling in *Drosophila*. 

A further proof of the importance of extracellular adenosine in the fly comes from the ablation of the *Drosophila* adenosine deaminase protein family through homologous recombination mutagenesis of all six *Adgf* genes [36]. Adenosine levels increased by these means cause not only larval and pupal death, as described above, but also degeneration of the larval fat body [23]. Moreover, increased adenosine in the larval hemolymph (a fluid plasma circulating throughout the interior body of insects and containing hemocytes for the production of hemocyanin, a molecule that transports oxygen) causes hyperglycemia mediated by AdoR activation, a mechanism that is conserved between *Drosophila* and mammals [38]. This hyperglycemic event further impairs the carbohydrate storage during the larval development and renders the flies very sensitive to the sugar in the diet, with potential deadly consequences. Adenosine signaling being associated with immune and stress responsescan thus have deleterious consequences on additional biological and/or pathological parameters.

There are further analogies between fruit flies and mammals regarding the adenosine/AdoR system. For instance, the steady-state extracellular concentration of adenosine is below the 300 nM range in the fly hemolymph, as in human blood [19]. High extracellular adenosine is cytotoxic for several cell types in both insects [28,29] and mammals. These features have justified the hypothesis that adenosine signaling represents evolutionarily well-conserved neuromodulation and homeostatic mechanisms in *Drosophila* and higher organisms.

### 4.3. Evidence from Exogenous ATP Receptors Expression

Although molecular and functional characterization of ionotropic P2X receptors is reported, for instance, in slime mold, green alga, and choanoflagellates, several invertebrates comprising *Drosophila* lack P2X-like genes, perhaps due to their loss at some point during evolution.

Moreover, the *Drosophila* genome reports only one NTDPase CD39-like gene, giving rise to four different transcripts, NTDPase-RA, NTDPase-RB, NTDPase-RC, and NTDPase-RD [39]. The protein encoded by NTDPase-RA expressed in Schneider 2 cells (a stable cell line derived from a macrophage-like lineage primary culture of late stage *Drosophila* embryos) indicates homology with mammalian NTDPase6, maximal activity obtained with GDP, IDP, and UDP as substrates; however, primary intracellular localization is in the endoplasmic reticulum [40]. The finding that the NTDPase-RA protein is not expressed on the cell surface is compatible with the absence of genes encoding for P2X ATP-gated cation channels and of protein homologs to P2X receptors in fruit flies [41]. Moreover, neither the presence nor the transport and accumulation of extracellular ADP or ATP have ever been quantified in *Drosophila* thus far.

By cross genome and multiple ligand/phylogenetic analysis of human and *Drosophila* G-coupled orphan receptors, some orthologues of the P2Y family have been identified in gene clusters of fruit flies containing nucleotide and lipid receptors. However, most members of these clusters are misnomers, not clustering with the conventional nucleotide/lipid receptors. A reason might be that uncommon nucleotides act as natural ligands, or they instead are not nucleotide receptors at all. Most importantly, the dataset of the considered sequences does not contain any representation from *Drosophila* GPCRs [42]. These results reinforce the assumption that P2X/P2Y signaling may not exist in the fruit fly.

While disappointing at first, the lack of extracellular nucleotides and subsequent signaling mediated by P2 receptors has instead rendered the fruit fly a very appealing assay platform wherein to scrutinize and dissect selected behaviors and functions driven by the expression of ATP receptors. We will now describe some among the most exemplifying studies. 

In *Drosophila*, the olfactory system is crucial for finding nutrients, eluding predators, and identifying mating partners. The mushroom bodies, consisting of a paired neuropil structure in the insect brain, are responsible for olfactory learning, memory, and multisensory integration, with different compartments controlling for instance aversive learning (hill) versus appetitive learning (medial tip). Via projecting neurons, the mushroom bodies receive olfactory information from the primary olfactory brain area, the antennal lobe, in turn receiving inputs directly from the olfactory sensory neurons in the antennae and mouth. Through the ectopic expression and activation of P2X2 receptor in the intrinsic neurons of the mushroom bodies (Kenyon cells), it was possible to establish a functional feedback from mushroom bodies to the antennal lobes in the olfactory pathway, thus suggesting a top-down modulation of olfactory information processing in *Drosophila* [43]. Moreover, dopaminergic signaling and release were measured after activation of P2X2 in mushroom bodies, with no regional or sex differences found in dopamine release [44].

Appropriate feeding behavior is crucial for survival in *Drosophila*. Ectopic expression of P2X2 in gustatory sensory neurons, and particularly in sweet-, bitter-, and water-sensing neurons committed to the sense of taste and feeding, has demonstrated the existence of independent processing of appetitive and aversive tastes, aimed at ensuring innate responses to appropriate feeding [45]. Moreover, heterologous expression of mammalian P2X2 in *Drosophila* taste neurons has expanded our knowledge of the structure–activity relationship of agonists’ action, through the rapid screening and identification of novel selective P2X2 agonists from an adenosine nucleotide library [46]. 

The giant fiber system of *Drosophila* controls motor neurons innervating the dorsal longitudinal flight musculature and consists of giant fiber neurons, two large interneurons in the brain with their targets in the thoracic ganglion, the tergotrochanteral muscle motor neuron, and the peripherally synapsing interneuron. By means of optically gated P2X2 ion channel activation in circumscribed groups of neurons, it was possible to provide noninvasive control of the connectivity and dynamics of neural circuits, of behavioral allocation to neurons, and of their activity patterns. In particular, photo-stimulation of transgenic P2X2 in neurons of the giant fiber system elicits the characteristic escape behaviors of jumping, wing beating, and flight. On the other hand, photo-stimulation of exogenously-expressed P2X2 in dopaminergic neurons causes changes in locomotor activity and locomotor patterns [47]. 

Taking further advantage of fly genetics, the isolated larval ventral nerve cord modified to express P2X2 in dopaminergic cells has allowed researchers to distinguish the functional dopamine releasable and reserve pools, with new dopamine synthesis playing a major part in long-term replenishment and uptake being more important for short-term dopamine replenishment [48]. A synoptic view of purinergic events occurring in *Drosophila* is reported in Table 1.

## 5. Future Challenges and Opportunities 

Purinergic signaling and *Drosophila* research represent two independent fields that have been much implemented in the past decades. An important yet relatively new topic with much promise is now the study of purinergic mechanisms in *Drosophila*. The present work thus has the aim of encouraging further research and an inclusive framework for understanding the extracellular role of purinergic signaling in *Drosophila*. In particular, we have shown here that despite the limited presence of a single AdoR for extracellular adenosine and only selected purinergic metabolic enzymes and transporters, fruit flies are nevertheless a versatile, scalable, and tunable model system for analyzing purinergic functional correlations, for restoring purinergic information corrupted by injury or disease, and for decoding the role not only of adenosine, as already described in several works, but especially of extracellular ATP binding to ectopically expressed P2 receptor subtypes. This topic constitutes a particularly expanding and challenging venue in *Drosophila* purinergic research, with high potential impact on pathophysiology, drug design, and preclinical testing. For instance, taste neurons in *Drosophila* might be a future opportunity for functional studies of neuronally expressed heteromeric P2X2/P2X3 or homomeric P2X3 receptors that are thought to act in nociceptive, inflammatory, and neuropathic pain. Likewise, P2X7 ectopically expressed either in glia, muscle, or motor neurons might be particularly suitable for decoding cell-dependent functional diversity and also for advancing the comprehension of a motor neuron disease, such as amyotrophic lateral sclerosis [49]. 

Although scientists are now more seriously working on these directions for developing new models, novel pharmacological, and genetic tools and providing continuous feedback on the impact of purinergic research in fruit flies, there are surely more twists and turns to navigate.

## Figures and Tables

**Table 1:** Synoptic view of purinergic events in *Drosophila melanogaster*. The table summarizes the impact of purinergic actions and mechanisms described in *Drosophila* cells and organs.

Effector	Biological Target and/or Function	Ref.
Adenosine	Fluid secretion from Malpighian tubules	[17]
Adenosine	Cytotoxic effect	[28,29]
Adenosine	Concentration below 300 nM in haemolymph	[19]
Adenosine	Hyperglycaemia in larval haemolymph	[38]
Adenosine	Synergistic signaling of AdoR, Ent2, Adgf-A	[37]
Adenosine deaminases	Stimulation of cell proliferation	[28,29]
ADGF-A	Null mutation is lethal in homozygosis	[23,36]
AdoR	*CG9753* gene identified as AdoR	[18]
AdoR	Endogenous brain expression and stress, immune, wake-cycle involvement	[22,23]
AdoR	*CG9753* homology with *ADORA2A*	[19]
AdoR	AdoR mutants are viable	[21]
AdoR	AdoR coupling to adenylate cyclase	[20]
AdoR	Activation of TNF, boosting of JunK	[25]
AdoR	Multipotent intestinal stem cell maintenance	[24]
Caffeine	cAMP increase, PKA activation	[21]
CNT1	Male infertility caused by mutations	[34]
ENT2	Nucleoside transporters genomic analysis	[26]
ENT2	Nucleoside transporter synaptic function, excitatory potentials increased by mutations	[27]
ENT2/AdoR	Amelioration of mutant Huntingtin-induced oxidative and heat stress response by knockdown	[35]
ENT2/CNT1/AdoR	Sequence homology with humans	[30]
ENT2/CNT1/AdoR	Rescue of imaginal discs cell deathby ablation	[33]
Ecto-5′-nucleotidases	Identification of five genes with alternative transcripts	[31]
NTDPase	Presence of just one CD39-like gene	[39]
NTDPase	Localization in the endoplasmic reticulum	[40]
P2X	Absence of P2X genes	[41]
P2Y	Absence of P2Y genes	[42]
**Effector**	**Function induced by ectopic expression**	**Ref.**
P2X2	Locomotor activity and patterns	[47]
P2X2	Olfactory information processing	[43]
P2X2	Appetitive and aversive taste	[45]
P2X2	Dopamine releasable and reserve pools	[48]
P2X2	Novel agonists identification	[46]
P2X2	Dopaminergic signaling in mushroom bodies	[44]

## Data Availability

Not applicable.

## Appendix A. Basic Notions about *Drosophila*

The “Species” *Drosophila melanogaster*, belonging to the “Genus” *Drosophila*, “Family” *Drosophilidae*, order “*Diptera*”, class “*Insecta*”, develops in a wide range of temperate habitats, being extremely dependent on temperature for survival and reproduction, and is also easily obtained in a laboratory environment. A chitinous exoskeleton covers the three main body segments of the fruit fly: the rounded head, the yellow-brown thorax, and the black-pigmented abdomen. The head includes a pair of short antennae, three small simple eyes (ocelli), plus two large red eyes, each formed by about 800 ommatidia that are structured within a precise and ordered geometry. The fruit fly possesses three pairs of segmented legs and a single pair of wings. *Drosophila* has a simplistic behavior. The flies are attracted by the smell of any food, but primarily plant materials; they are sensitive to slight differences in air currents and light intensity, with the tendency to escape when shadows or movements are perceived. *Drosophila* mates extensively among the opposite sex. For over a century, due to low cost, easy handling, and high abundance, fruit flies have been considered indispensable organisms for research. Not surprisingly, back in 1910, experiments with *Drosophila* laid the foundation for the chromosomal theory of inheritance [50].

## Appendix B. Basic Notions about Purinergic Transporters, Ectonucleotidases, and Receptors

**The extracellular release of purine/pyrimidine molecules** comprising the most diffuse ATP is an overtime mutable event mediated by purinergic transporters and occurring particularly in secretory cells via vesicular exocytosis or non-exocytotic connexin-43, pannexin-1 pathways. Purine and pyrimidine release can also occur by facilitated diffusion through concentrative and equilibrative nucleotide-specific transporters by electro-diffusional transport through ion channels and, finally, via lytic mechanisms in all kinds of damaged or dying cells.

**Ectonucleotidases** generate ADP, AMP, UDP, UMP, and adenosine by hydrolyzing nucleoside 5′-tri-, 5′-di-, and 5′-mono-phosphates. Four major families of ectonucleotidases exist: ectonucleoside triphosphate diphosphohydrolases (E-NTPDase, hydrolyzing only extracellular nucleoside tri- and di-phosphates), ectonucleotide pyrophosphatase/phosphodiesterase (E-NPP, breaking up pyrophosphate 5′-monodiester bonds), ecto-5′-nucleotidase (CD73, hydrolyzing only nucleosides monophosphates), and alkaline phosphomonoesterase (AP, releasing inorganic phosphate from nucleoside 5′-tri, -di, and -monophosphates).

**P1 receptors** are G-protein coupled receptors, in turn classified into four subtypes (A1, A2A, A2B, and A3) and encoded by different genes. A1 and A3 are receptors coupled to Gi/Go/q proteins, thus leading to inhibition of cAMP synthesis and activation of phospholipase C. A2A and A2B are instead coupled to Gs proteins, thus leading to increased cAMP synthesis.

**Ionotropic P2X receptors** subclassified into seven different subtypes (P2X1-7) are non-selective Ca^2+^, Na^+^, and/or K^+^ channels, responsive exclusively to ATP. P2X receptors are ubiquitously expressed on cells as monomers, or omo- and hetero-trimers or hexamers.

**Metabotropic P2Y receptors** are classified into eight distinct subtypes (P2Y1, P2Y2, P2Y4, P2Y6, P2Y11, P2Y12, P2Y13, and P2Y14) and are activated by ATP, ADP, UTP, UDP, and UDP-glucose. They mediate slow responses. In general, P2Y1, P2Y2, P2Y4, and P2Y6 are positively coupled to PLC, via Gq/11 proteins, while P2Y12, P2Y13, and P2Y14 are negatively coupled to adenylyl cyclase via Gi proteins.
